# A novel E-cadherin/SOX9 axis regulates cancer stem cells in multiple myeloma by activating Akt and MAPK pathways

**DOI:** 10.1186/s40164-022-00294-x

**Published:** 2022-07-13

**Authors:** Parinya Samart, Yon Rojanasakul, Surapol Issaragrisil, Sudjit Luanpitpong

**Affiliations:** 1 Department of Immunology, Faculty of Medicine Siriraj Hospital, Mahidol University, Bangkok, Thailand; 2 Siriraj Center of Excellence for Stem Cell Research, Faculty of Medicine Siriraj Hospital, Mahidol University, 2 Siriraj Hospital, Bangkoknoi, Bangkok, 10700 Thailand; 3 WVU Cancer Institute, Department of Pharmaceutical Sciences, West Virginia University, Morgantown, WV USA; 4 Division of Hematology, Department of Medicine, Faculty of Medicine Siriraj Hospital, Mahidol University, Bangkok, Thailand; 5 Bangkok Hematology Center, Wattanosoth Hospital, BDMS Center of Excellence for Cancer, Bangkok, Thailand

**Keywords:** Multiple myeloma, E-cadherin, SOX9, Cancer stem cells, Self-renewal

## Abstract

Cancer stem cells (CSCs) have been identified in multiple myeloma (MM) and are widely regarded as a key driver of MM initiation and progression. E-cadherin, in addition to its established role as a marker for epithelial-mesenchymal transition, also plays critical roles in controlling the aggressive behaviors of various tumor cells. Here, we show that depletion of E-cadherin in MM cells remarkably inhibited cell proliferation and cell cycle progression, in part through the decreased prosurvival CD138 and Bcl-2 and the inactivated Akt and MAPK pathways. CSC features, including the ability of the cells to form clonogenic colonies indicative of self-renewal and side population, were greatly suppressed upon the depletion of E-cadherin and subsequent loss of SOX9 stem-cell factor. We further provide evidence that SOX9 is a downstream target of E-cadherin-mediated CSC growth and self-renewal—ectopic re-expression of SOX9 in E-cadherin-depleted cells rescued its inhibitory effects on CSC-like properties and survival signaling. Collectively, our findings unveil a novel regulatory mechanism of MM CSCs via the E-cadherin/SOX9 axis, which could be important in understanding the long-term cell survival and outgrowth that leads to relapsed/refractory MM.

**Supplementary Information:**

The online version contains supplementary material available at 10.1186/s40164-022-00294-x.

## To the editor,

Novel therapies for multiple myeloma (MM), such as proteasomal inhibitors, immunomodulatory drugs, and CAR-T cell therapy, have improved palliation and response rates, providing a longer disease-free period; however, MM inevitably progresses in the vast majority of patients [1]. Cancer stem cells (CSCs), also known as tumor initiating cells, are believed to be the root cause of tumor recurrence for most if not all malignancies, including MM [2]. Identification of molecular pathways that contribute to CSCs is essential to understanding how MM progression is regulated. E-cadherin (encoded by *CDH1*) is known to have a pivotal role in the regulation of embryonic and normal adult stem cell survival and self-renewal [3, 4]. In solid tumors, loss of E-cadherin has traditionally been viewed as a hallmark of the occurrence of epithelial-to-mesenchymal transition, linking to metastasis. The role of E-cadherin in solid tumor growth, however, remains controversial and appears to be cell type- and tumor stage-dependent [5, 6]. E-cadherin protein level is significantly higher in MM tissues compared to normal tissues [7], and its increased mRNA expression has been correlated with symptomatic MM [8] and plasma cell leukemia, an aggressive variant of MM (Additional file 2: Figure S1). We have previously reported the decreased E-cadherin level in poorly disseminated MM cells mediated by hyper-*O*-GlcNAcylation [9].

**Fig. 1:**
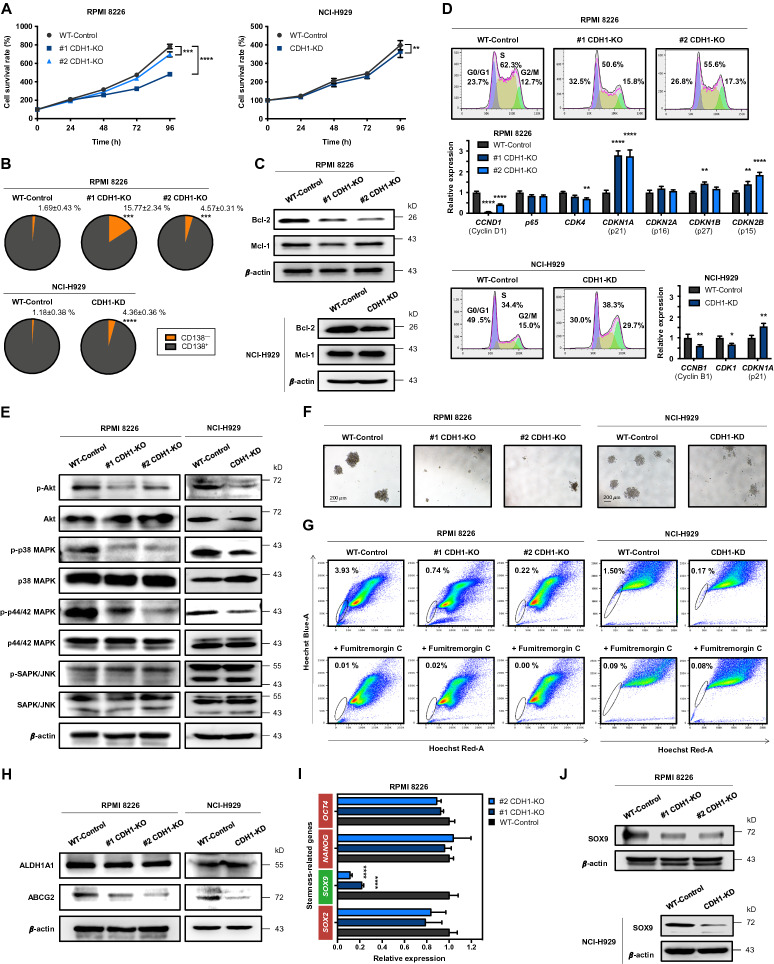
E-cadherin regulates cell growth and CSC-like phenotypes in human MM-derived cells. E-cadherin was depleted in RPMI 8226 and NCI-H929 cells using the CRISPR/Cas9 system, designated as CDH1-KO RPMI 8226 and CDH1-KD NCI-H929 cells, respectively (Additional file 2: Figure S2). **A** Cell viability was evaluated by MTT assay to monitor cell proliferation at 24, 48, 72, and 96 h of culture. Data are mean ± SD (*n* = 3). ^**^p < 0.01, ^***^p < 0.001, ^****^p < 0.0001 versus WT control cells; two-tailed Student’s *t*-test. **B** Cell surface expression of CD138 was analyzed by flow cytometry. The proportion of CD138-positive (CD138^+^) and CD138-negative (CD138^−^) cells is shown. Data are mean ± SD (*n* = 3). ^***^p < 0.001, ^****^p < 0.0001 versus WT cells; two-tailed Student’s *t*-test. **C** Western blot analysis of prosurvival Bcl-2 and Mcl-1 proteins. β-actin was used as a loading control. The significant decrease in Bcl-2, but not Mcl-1, level was detected in CDH1-KO RPMI 8226 and CDH1-KD NCI-H929 cells compared to WT cells (^**^p < 0.01; two-tailed Student’s *t*-test). **D** (upper) Cell cycle analysis based on DNA content was analyzed by flow cytometry using propidium iodide staining. (lower) Quantitative real-time PCR (RT-qPCR) analysis of mRNA expression of cell cycle regulator genes. *GAPDH* served as the internal control. Data are mean ± SD (*n* = 3). ^*^p < 0.05, ^**^p < 0.01, ^****^p < 0.0001 versus WT cells; two-tailed Student’s *t*-test. **E** Western blot analysis of Akt and MAPK family proteins. The significant decrease in phosphorylated (p)-Akt, p-p38, and p-p44/42 levels was detected in CDH1-KO RPMI 8226 and CDH1-KD NCI-H929 cells compared to WT cells (^*^p < 0.05; two-tailed Student’s *t*-test). **F** Representative micrographs showing MM colonies under clonogenic assay (see also Additional file 2: Figure S3 for quantitative analysis of colony number and size). Scale bar = 200 μm. **G** SP subpopulation analysis using flow cytometry based on Hoechst 33342 dye efflux. SP cells (*box*) were determined by their disappearance in the presence of fumitremorgin C (see also Additional file 2: Figure S5 for quantitative analysis). **H** Western blot analysis of ALDH1A1 and ABCG2. A significant decrease in ABCG2, but not ALDH1A1, level was detected in CDH1-KO RPMI 8226 and CDH1-KD NCI-H929 cells compared to WT cells (^*^p < 0.05; two-tailed Student’s *t*-test). **I** RT-qPCR analysis of mRNA expression of stemness-regulated genes. Data are mean ± SD (*n* = 3). ^****^p < 0.0001 versus WT cells; two-tailed Student’s *t*-test. **J** Western blot analysis of SOX9 level in CDH1-KO RPMI 8226 and CDH1-KD NCI-H929 cells (see also Additional file 2: Figure S6 for quantitative analysis)

CSC phenotypes include their self-renewal and proliferative properties. To investigate the functional role of E-cadherin in regulating MM CSCs, we first established E-cadherin-depleted cells in human MM-derived cell lines RPMI 8226 and NCI-H929 using the CRISPR/Cas9 system (Additional file 2: Figure S2) and examined its effects on cell growth and cell cycle. Detailed methods can be found in Additional file 1. Figure 1A − C shows that both E-cadherin-depleted cells were less proliferative than wild type (WT) control cells, corresponding to the increased CD138-negative subpopulation and decreased prosurvival Bcl-2, but not Mcl-1. Our findings were consistent with a previous study reporting the prosurvival effect of CD138 in MM [10]. We also found that loss of E-cadherin caused either G0/G1 or G2/M arrest, depending on the cellular context, by controlling its key cell cycle regulators in each phase (Fig. 1D). The PI3K/Akt and MAPK pathways have been reported to regulate the proliferation and survival of MM cells [11]. Herein, we showed that E-cadherin activates Akt, p38, and p44/42 (ERK1/2), but not SAPK/JNK, via protein phosphorylation (Fig. 1E). Altogether, these results support the positive regulatory role of E-cadherin in MM cell growth and survival.

**Fig. 2:**
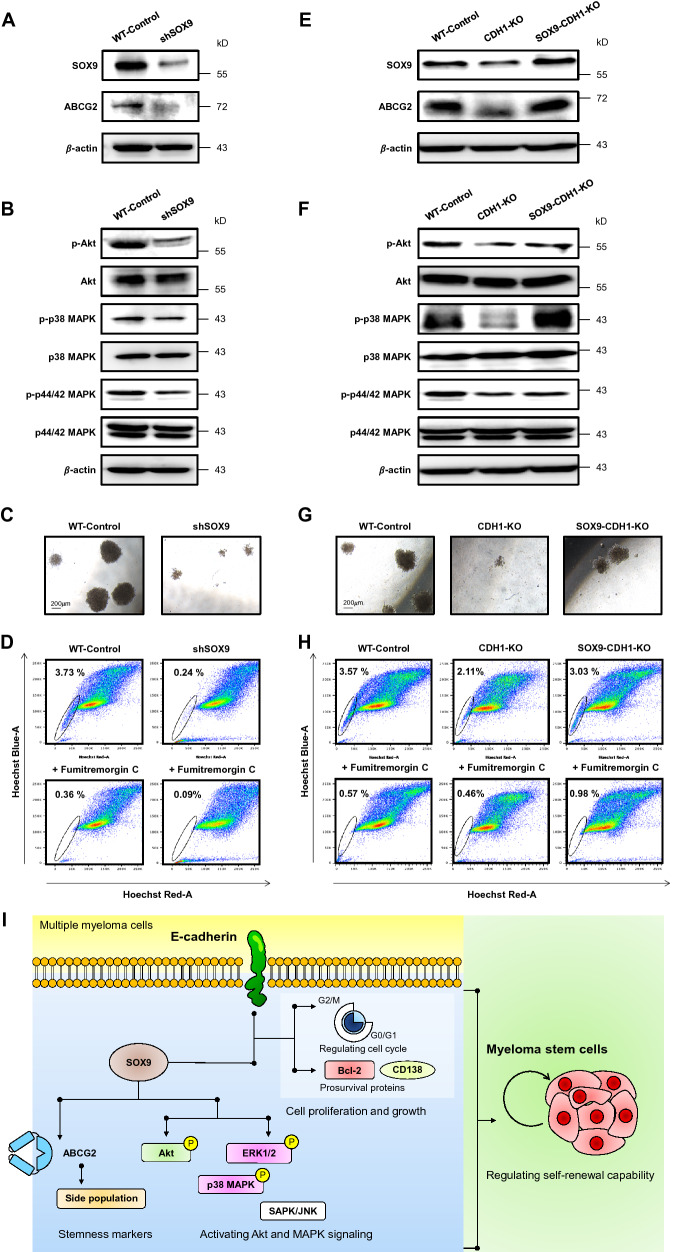
E-cadherin/SOX9 axis regulates CSCs in human MM-derived cells. **A–D** SOX9 was depleted in RPMI 8226 cells expressing high endogenous SOX9 using lentiviral particles carrying shSOX9 or non-target sequence (WT-control). **A** Western blot analysis of SOX9 and ABCG2 levels. β-actin was used as a loading control. The significant decrease in SOX9 and ABCG2 levels was detected in shSOX9 cells compared to WT cells (^**^p < 0.01; two-tailed Student’s *t*-test). **B** Western blot analysis of Akt and MAPK family proteins. The significant decrease in p-Akt, p-p38, and p-p44/42 levels was detected in shSOX9 cells compared to WT cells (^*^p < 0.05; two-tailed Student’s *t*-test). **C** Representative micrographs showing MM colonies under clonogenic assay (see also Additional file 2: Figure S7 for quantitative analysis). Scale bar = 200 μm. **D** SP analysis using flow cytometry based on Hoechst 33342 dye efflux (see also Additional file 2: Figure S8 for quantitative analysis). **E–H** Rescue experiments were performed in CDH1-KO RPMI 8226 cells by transfection of the cells with SOX9 plasmid. Cells with SOX9 restoration were designated SOX9-CDH1-KO cells. **E** Western blot analysis of SOX9 and ABCG2 levels. β-actin was used as a loading control. The significant increase in SOX9 and ABCG2 levels was detected in SOX9-CDH1-KO cells compared to CDH1-KO cells (see also Additional file 2: Figure S9 for quantitative analysis). **F** Western blot analysis of Akt and MAPK family proteins. The significant increase in p-Akt, p-p38, and p-p44/42 levels was detected in SOX9-CDH1-KO cells compared to CDH1-KO cells (see also Additional file 2: Figure S10 for quantitative analysis). **G** Representative micrographs showing MM colonies under clonogenic assay (see also Additional file 2: Figure S11 for quantitative analysis). Scale bar = 200 μm. **H** SP analysis using flow cytometry based on Hoechst 33342 dye efflux (see also Additional file 2: Figure S12 for quantitative analysis). **I** Schematic illustration of how E-cadherin/SOX9 axis governs cell growth and self-renewal of CSCs, in part via Akt and MAPK signaling, in MM cells. It is worth noting that other molecules might be involved in this regulatory axis, which requires further investigation

We hypothesized that E-cadherin may be involved in CSC self-renewal. To investigate, colony-forming ability, the potential of a single cell to indefinitely grow and survive [12], was evaluated by clonogenic assay. Figure 1F shows that depletion of E-cadherin resulted in a reduction in both the number and size of MM colonies when compared to WT cells (Additional file 2: Figure S3), which could be reactivated by the restoration of E-cadherin (Additional file 2: Figure S4). Additionally, we found that depletion of E-cadherin reduced the side population (SP) phenotype, a common feature of CSCs related to the ABCG2 multidrug efflux transporter (Fig. 1G and H; Additional file 2: Figure S5). Profiling of stemness-related genes, i.e., *SOX2*, *SOX9*, *NANOG*, and *OCT4*, pointed out that SOX9 could be a key regulator of E-cadherin-mediated MM CSCs (Fig. 1I and J; Additional file 2: Figure S6). To first test whether SOX9 is functionally linked to CSCs, SOX9 was depleted in RPMI 8226 cells using shRNA. Similar to E-cadherin, depletion of SOX9 reduced Akt and MAPK activity, colony-forming capacity, and SP cells and its corresponding ABCG2 when compared to WT cells (Fig. 2A − D; Additional file 2: Figures S7 and S8), indicating the critical role of SOX9 in MM CSCs. To further validate that SOX9 is downstream of E-cadherin, rescue experiments were conducted in which SOX9 plasmid was ectopically overexpressed in E-cadherin-depleted cells. Figure 2E and F shows that the reduced SOX9 and ABCG2 as well as the reduced Akt and MAPK signaling in E-cadherin depleted cells could be rescued by ectopic SOX9 (see also Additional file 2: Figures S9 and S10). This SOX9 restoration also reversed the inhibitory effects of E-cadherin depletion on the colony forming capacity and SP cells (Fig. 2G and H; Additional file 2: Figures S11 and S12), thus confirming that E-cadherin mediates MM CSCs via SOX9. We also found that SOX9 is, in turn, necessary for maintaining E-cadherin level (Additional file 2: Figure S13), indicating a positive feedback loop that controls MM CSCs.

In summary, we revealed a novel regulatory mechanism of MM CSCs via the E-cadherin/SOX9 axis (Fig. 2I), which could be important in understanding the long-term cell survival and outgrowth that leads to relapsed/refractory MM. Our findings provided a potential rationale for targeting E-cadherin/SOX9 axis, while in vivo studies are warranted to further validate this hypothesis.

## Supplementary Information

**Additional file 1: **Detailed methods.

**Additional file 2: Figure S1.** Analysis of CDH1 mRNA expression in clinical samples using publicly available microarray data. **Figure S2.** Successful depletion of E-cadherin by the CRISPR/Cas9 system in the human MM– derived cell lines RPMI 8226 and NCI-H929. **Figure S3.** Depletion of E-cadherin inhibits the clonogenic potential of MM cells. **Figure S4.** Restoration of Ecadherin into E-cadherin-depleted cells rescues the clonogenic potential of MM cells. **Figure S5.** Depletion of E-cadherin decreases the proportion of the SP subpopulation in MM cells. **Figure S6.** Depletion of E-cadherin suppresses SOX9 level in MM cells. **Figure S7.** Depletion of SOX9 reduces the clonogenic potential of MM cells. **Figure S8.** Depletion of SOX9 decreases the proportion of SP subpopulation in MM cells. **Figure S9.** Re-expression of SOX9 in E-cadherin-depleted MM cells rescues the ABCG2 level. **Figure S10.** Re-expression of SOX9 in E-cadherin-depleted MM cells reactivates Akt and MAPK signaling. **Figure S11.** SOX9 regulates E-cadherin-mediated clonogenic growth in MM cells. **Figure S12.** Re-expression of SOX9 induces the acquisition of the SP subpopulation in E-cadherin-depleted MM cells. **Figure S13.** Depletion of SOX9 suppresses E-cadherin level in MM cells.

## Data Availability

The datasets generated and/or analyzed during the current study are available from the corresponding author on reasonable request. Additional file information is available in Additional files 1 and 2.

## Abbreviations

CSC: Cancer stem cells
MM: Multiple myeloma
p-: Phosphorylated
RT-qPCR: Quantitative real-time PCR
SP: Side-population
WT: Wild type
