# Supplementary material for: A novel E-cadherin/SOX9 axis regulates cancer stem cells in multiple myeloma by activating Akt and MAPK pathways
Source: Exp Hematol Oncol. 2022 Jul 13;11:41. doi: 10.1186/s40164-022-00294-x (PMC9277902; doi:10.1186/s40164-022-00294-x)
Supplement: Supplementary file 1 — Additional file 1: Detailed methods. [file 40164_2022_294_MOESM1_ESM.pdf]

Additional file 1: Detailed methods

Reagents and antibodies

3-(4,5-Dimethyl-2-thiazolyl)-2,5-diphenyl-2H-tetrazolium bromide (MTT) and antibody against β-actin conjugated to peroxidase (#A3854) were obtained from Sigma-Aldrich (St. Louis, MO, USA). ABCG2 inhibitor fumitremorgin C (FTC) was obtained from EMD Biosciences (San Diego, CA, USA). Mouse FITC-conjugated anti-human CD138 (Syndecan-1) antibody (#356508) was obtained from BioLegend (San Diego, CA, USA). Rabbit anti-human E-cadherin (#3195), ALDH1A1 (#12035), ABCG2 (#4477), p-Akt (#4060), Akt (#9272), p-p38 MAPK (#4511), p38 MAPK (#8690), p-p44/42 MAPK (#4370), p44/42 MAPK (#4695), p-SAPK/JNK (#4668), and SAPK/JNK (#9252) antibodies were purchased from Cell Signaling Technology (Beverly, MA, USA). Rabbit anti-human SOX9 antibody (#AB5535) and secondary antibody (#AP132P) were from EMD Millipore (Burlington, MA, USA). For immunofluorescence, anti-rabbit IgG-Alexa Fluor 488 (#A11008) was from Life Technologies (Eugene, OR, USA). All gene-specific primers were synthesized by Integrated DNA Technologies (Coralville, IA, USA).

Cell culture

Human MM-derived cell lines RPMI 8226 and National Cancer Institute (NCI)-H929 cells were purchased from the American Type Culture Collection (ATCC; Manassas, VA, USA) and cultured in RPMI 1640 medium supplemented with 10% fetal bovine serum (FBS) and 1% penicillin/streptomycin at 37 °C, 5% CO_2, 95% humidity, while HEK293T cells were from ATCC and maintained in DMEM-high glucose medium containing 10% FBS without antibiotics. Mycoplasma negativity was routinely tested by using MycoAlert™ PLUS Mycoplasma Detection Kit (Lonza, Cologne, Germany). Peripheral blood mononuclear cells

(PBMC) obtained from healthy donors after informed consent and after approval by the Siriraj Institutional Review Board (COA No. Si 101/2015) were used as normal control cells.

CRISPR/Cas9 system

pLentiCRISPRv2 plasmids containing guide RNA (gRNA) targeting *CDH1* were purchased from Genscript (Piscataway, NJ, USA). gRNA sequences targeting *CDH1* are CCTCGACACCCGATTCAAAG. Empty pLentiCRISPRv2 vector was used as control. Lentiviral particles were generated in HEK293T cells by co-transfection with pCMV-dR8.2 dvpr packaging (Addgene #8455) and pCMV-VSV-G envelope vectors (Addgene #8454) using Lipofectamine 3000 (Invitrogen, Carlsbad, CA, USA). RPMI 8226 and NCI-H929 cells were transduced with the virus in the presence of 8 μg/mL hexadimethrine bromide (polybrene), selected with puromycin (2 μg/mL) and assessed for gene knockdown efficiency using western blot analysis and immunofluorescence. Target regions of DNA were amplified by polymerase chain reaction (PCR) and sequenced using ABI PRISM BigDye Terminator Cycle Sequencing Kit v3.1 (Applied Biosystems, Waltham, MA, USA) performing by 1st BASE (Singapore). Sequence analysis was performed by using MultAlin software.

Genetic manipulation of SOX9

Lentiviral viral particles carrying short hairpin RNA sequence against human *SOX9* (shSOX9) (Addgene #40644) were used to knockdown SOX9 expression in RPMI 8226 cells. Non-effective, scrambled shRNA in lentiviral vector (#TR30021; Origene, Rockville, MD, USA) was used to produce control particles. For rescue experiments, CDH1-KO RPMI 8226 cells were transfected with scramble or SOX9 overexpression plasmid (Origene) using Lipofectamine 3000. The transfected cells were allowed to recovery for 48 h and SOX9 level was evaluated by western blot analysis using SOX9 antibody before each experiment.

Cell proliferation assay

Cell proliferation was performed by using MTT colorimetric assay. Cells were plated at the density of 5,000 cells per well in 96-well flat-bottomed microplates and cell viability was determined at 0, 24, 48, 72, and 96 h. A total of 10 μL per well of a 5 mg/mL solution of MTT in phosphate-buffered saline (PBS) was added and incubated for 4 h at 37 °C. Subsequently, 100 μL solubilizing buffer containing 10% SDS in 0.01 M HCl was added to each well and plates were incubated overnight at 37 °C to allow complete solubilization of the purple formazan crystals. Absorbance of the colored product was then measured at a wave length of 570 nm using a microplate reader (Synergy™ H1, BioTek Instruments, Winooski, VT, USA). Background was subtracted.

Cell cycle analysis

Cells were plated in 6-well plates at density of 1×10^6 cells per mL and incubated in serum-free medium for 24 h. Cells were then incubated in the complete medium for 24 h, after which the cell cycle analysis was performed using CycleTEST™ PLUS DNA reagent kit (BD Biosciences, San Jose, CA, USA) according to the manufacturer's instructions. For each cell population, 10,000 cells were analyzed by BD FACS CantoII flow cytometer (BD Biosciences) and the proportion in G0/G1, S, and G2/M phases were analyzed by using FlowJo software.

Side population (SP) analysis

Cells at a density of 1×10^6 cells per mL were labeled with 10 μg/mL Hoechst 33342 (Invitrogen, Eugene, OR, USA) in RPMI 1640 medium supplemented with 10% FBS at 37 °C for 90 min with rotation. As a control, an aliquot of each sample was treated with 25 μM ABCG2 inhibitor FTC for 10 minutes at room temperature, prior to the addition of Hoechst

33342. At the end of the incubation, cells were washed and resuspended in ice-cold DPBS without calcium and magnesium supplemented with 2% FBS and 10 mM HEPES. Cells were then filtered through a 70-μm filter to obtain a single cell suspension. Dead cells were excluded on the basis of 2 μg/mL propidium iodide (Life Technology, Eugene, OR, USA) incorporation before analysis. SP analysis was performed using BD FACSAria™ Fusion cell sorter (BD Biosciences) using near-UV laser and Hoechst Blue (450/20) and Red (670 LP) filters. SP fraction was calculated based on the disappearance of SP cells in the presence of FTC. The data were analyzed using FlowJo software.

Clonogenic assay

Clonogenic growth assay was performed as previously described using methylcellulose (MC)-based medium (MethoCult™ H4100, StemCell Technologies, Vancouver, Canada) with slight modifications [1]. Briefly, cells at a concentration of 200 cells per 500 μL were cultured in 1% MC supplemented with 30% FBS in 24-well plates for 14–21 days. Colonies were imaged by an inverted microscope on an Eclipse Ti-U instrument (Nikon, Tokyo, Japan) and colonies consisting of more than 40 cells were counted as one positive colony. Colony size was measured by area using NIS-Element D software. Representative whole plate images of colonies were photographed using a Canon EOS 700D.

Western blot analysis

Cells were lysed in protein lysis buffer (Cell Signaling Technology, Danvers, MA, USA) containing protease inhibitors cocktail (Roche Diagnostics, Mannheim, Germany) and protein concentration was measured using BCA assay kit (Thermo Fisher Scientific, Rockford, IL, USA). A total protein of 30–60 μg was subjected to SDS-PAGE electrophoresis. After transfer to PVDF membranes, indicated primary antibodies and HRP-linked secondary antibodies were

incubated with the membrane for overnight at 4 °C and 2 h at room temperature, respectively. The membrane was detected by ECL reagent (EMD Millipore) and imaged using a digital imaging system (ImageQuant LAS 4010, GE Healthcare Bio-Sciences AB, Uppsala, Sweden). β-actin was used as a loading control. Band intensity values were semi-quantified using Image J software.

Quantitative real-time PCR (RT-qPCR)

Total RNA was extracted from cells using Trizol reagent (Molecular Research Center, Cincinnati, OH, USA) and quantified using NanoDrop 2000 spectrophotometer (Thermo Fisher Scientific, Wilmington, DE, USA). cDNAs were synthesized with SuperScript III and oligo (dT) primers (Thermo Fisher Scientific, Vilnius, Lithuania) according to the manufacturer's protocols. RT-qPCR was carried out on CFX96 Touch™ Real-Time PCR Detection System (Bio-Rad, Hercules, CA, USA) with SYBR™ Select Master Mix (Thermo Fisher Scientific, Vilnius, Lithuania) using gene-specific primers. Relative mRNA expression was quantified using the $2^{-\Delta\Delta CT}$ method with *GAPDH* as a housekeeping gene.

Flow cytometry

Cell surface expression of CD138 was detected in single cell suspensions using FITC-conjugated anti-human CD138 antibody. Staining was performed for 15 min at room temperature, after which fluorescence intensity was acquired using BD FACS CantoII flow cytometer. The data were analyzed using FlowJo software.

Immunofluorescence (IF)

Cells at a density of 30,000 cells per 300 μL were adhered onto glass slides using a Cytospin centrifuge (Cytospin™ 4 Cytocentrifuge, Thermo Fisher Scientific, Waltham, MA, USA).

Cells were fixed with 4% paraformaldehyde (PFA) for 15 min and block with 5% bovine serum albumin (BSA) in PBS for 2 h at room temperature. Cytospinned slides were stained with anti-E-cadherin at 4 °C overnight and then with anti-rabbit IgG-Alexa Fluor 488 for 2 h at room temperature. Subsequently, slides were washed with PBS. Slides were counterstained with DAPI for nucleus detection using mounting medium with DAPI (Ibidi, Martinsried, Germany) and visualized under fluorescence microscope (Eclipse Ti-U).

Statistical analysis

Data represent means ± SD from at least three independent experiments. Statistical analysis was performed by two-sided, unpaired Student's *t*-test or one-way ANOVA followed by Tukey's multiple comparison test at a significance level of $p < 0.05$ (GraphPad Prism, San Diego, CA, USA).

Supplementary references

1. Lauring J, Abukhdeir AM, Konishi H, Garay JP, Gustin JP, Wang Q, et al. The multiple myeloma associated MMSET gene contributes to cellular adhesion, clonogenic growth, and tumorigenicity. Blood. 2008;111(2):856–64.
