# Supplementary material for: A novel E-cadherin/SOX9 axis regulates cancer stem cells in multiple myeloma by activating Akt and MAPK pathways
Source: Exp Hematol Oncol. 2022 Jul 13;11:41. doi: 10.1186/s40164-022-00294-x (PMC9277902; doi:10.1186/s40164-022-00294-x)
Supplement: Supplementary file 2 — Additional file 2: Figure S1. Analysis of CDH1 mRNA expression in clinical samples using publicly available microarray data. Figure S2. Successful depletion of E-cadherin by the CRISPR/Cas9 system in the human MM– derived cell lines RPMI 8226 and NCI-H929. Figure S3. Depletion of E-cadherin inhibits the clonogenic potential of MM cells. Figure S4. Restoration of Ecadherin into E-cadherin-depleted cells rescues the clonogenic potential of MM cells. Figure S5. Depletion of E-cadherin decreases the proportion of the SP subpopulation in MM cells. Figure S6. Depletion of E-cadherin suppresses SOX9 level in MM cells. Figure S7. Depletion of SOX9 reduces the clonogenic potential of MM cells. Figure S8. Depletion of SOX9 decreases the proportion of SP subpopulation in MM cells. Figure S9. Re-expression of SOX9 in E-cadherin-depleted MM cells rescues the ABCG2 level. Figure S10. Re-expression of SOX9 in E-cadherin-depleted MM cells reactivates Akt and MAPK signaling. Figure S11. SOX9 regulates E-cadherin-mediated clonogenic growth in MM cells. Figure S12. Re-expression of SOX9 induces the acquisition of the SP subpopulation in E-cadherin-depleted MM cells. Figure S13. Depletion of SOX9 suppresses E-cadherin level in MM cells. [file 40164_2022_294_MOESM2_ESM.pdf]

Additional file 2: Figures S1–S13.

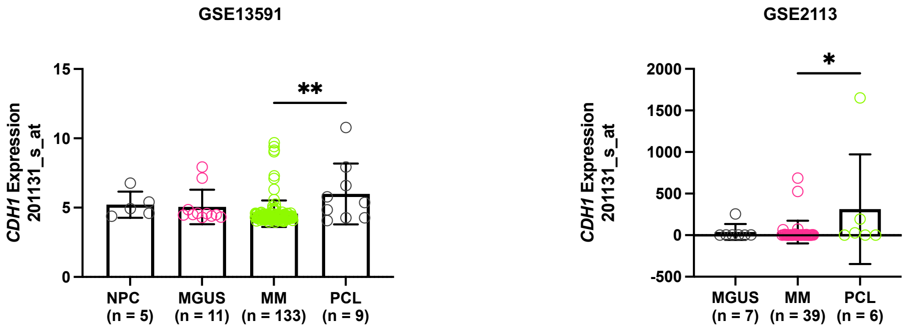

Fig. S1 Analysis of *CDH1* mRNA expression in clinical samples using publicly available microarray data. The data were obtained from Gene Expression Omnibus (GEO) under accession numbers GSE13591 and GSE2113. Scatter plots of each value are shown along with the mean ± SD. $^{*}p < 0.05$, $^{**}p < 0.01$ versus MM; one-way ANOVA with Tukey's multiple comparison test. NPC: normal plasma cells; MGUS: monoclonal gammopathy of undetermined significance; MM: multiple myeloma; PCL: plasma cell leukemia.

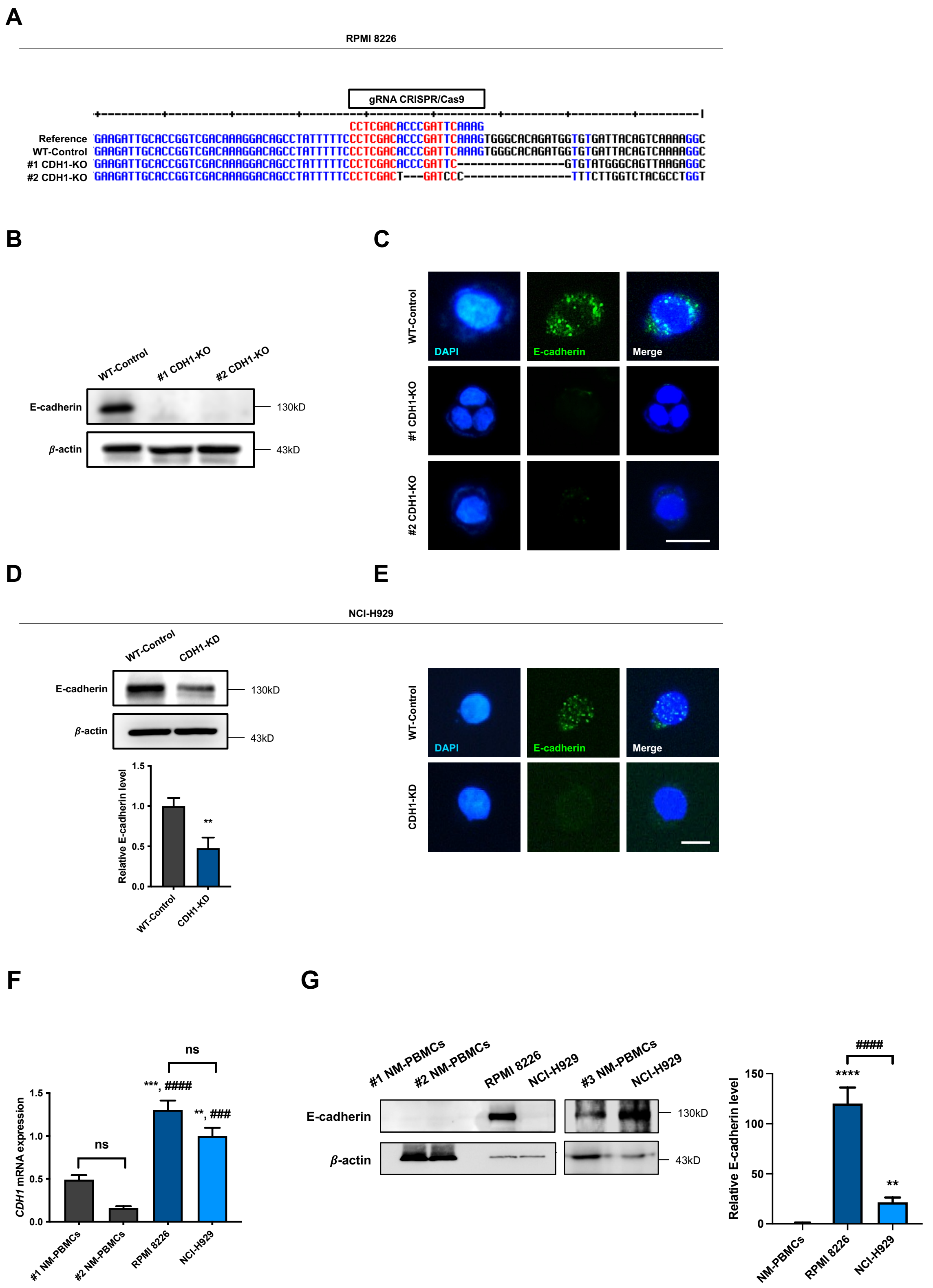

Fig. S2 Successful depletion of E-cadherin by CRISPR/Cas9 system in human MM-derived cell lines RPMI 8226 and NCI-H929 cells. **A** Sequence analysis of CRISPR/Cas9 target *CDH1* mutations by alignment based on NCBI reference sequences of NM_004360.5 in MultAlin

software with indicated gRNA sequences. Black dashes (-) represent deletion. **B** Western blots showing that the CDH1-gRNA effectively creates E-cadherin knockout (CDH1-KO) RPMI 8226 cells when compared to Cas9 control (WT-control) cells. β-actin was used as a loading control. **C** E-cadherin on plasma membrane was assessed by immunofluorescence staining for E-cadherin (green) in non-permeabilized cells. Nuclei were counterstained with DAPI (blue). Scale bar = 20 μm. #1 and #2 represent CDH1-KO clone number 1 and 2, respectively. **D** Western blots showing that CDH1-gRNA remarkably decreases the protein level of E-cadherin in E-cadherin knockdown (CDH1-KD) NCI-H929 cells when compared to WT cells. β-actin was used as a loading control. (lower) Quantitative analysis of E-cadherin level after normalization to β-actin and relative to WT cells. Data are mean ± SD ($n = 3$). $^{**}p < 0.01$ versus WT-control cells; two-tailed Student's *t*-test. **E** Immunofluorescence staining assay demonstrating the decrease in E-cadherin protein (green) on the plasma membrane of non-permeabilized CDH1-KD NCI-H929 cells when compared to WT cells. Nuclei were counterstained with DAPI (blue). Scale bar = 20 μm. Notably, E-cadherin is overexpressed in RPMI 8226 and NCI-H929 cells when compared to peripheral blood mononuclear cells obtained from healthy donors (NM-PBMCs), at both mRNA and protein levels. **F** RT-qPCR analysis of *CDH1* mRNA expression. Expression levels were normalized to housekeeping *GAPDH* and relative to NCI-H929 cells. Data are mean ± SD ($n = 3$). $^{**}p < 0.01$, $^{***}p < 0.001$ versus case #1 NM-PBMCs; $^{\#\#\#}p < 0.001$, $^{\#\#\#\#}p < 0.0001$ versus case #2 NM-PBMCs; one-way ANOVA with Tukey's multiple comparison test. ns, not significant. **G** Western blot analysis of E-cadherin level. (left) Representative immunoblots are shown. (right) Quantitative analysis using densitometry, after normalization to β-actin. Data are mean ± SD ($n = 3$). $^{**}p < 0.01$, $^{****}p < 0.0001$ versus NM-PBMCs; $^{\#\#\#\#}p < 0.0001$ versus RPMI 8226 cells; one-way ANOVA with Tukey's multiple comparison test.

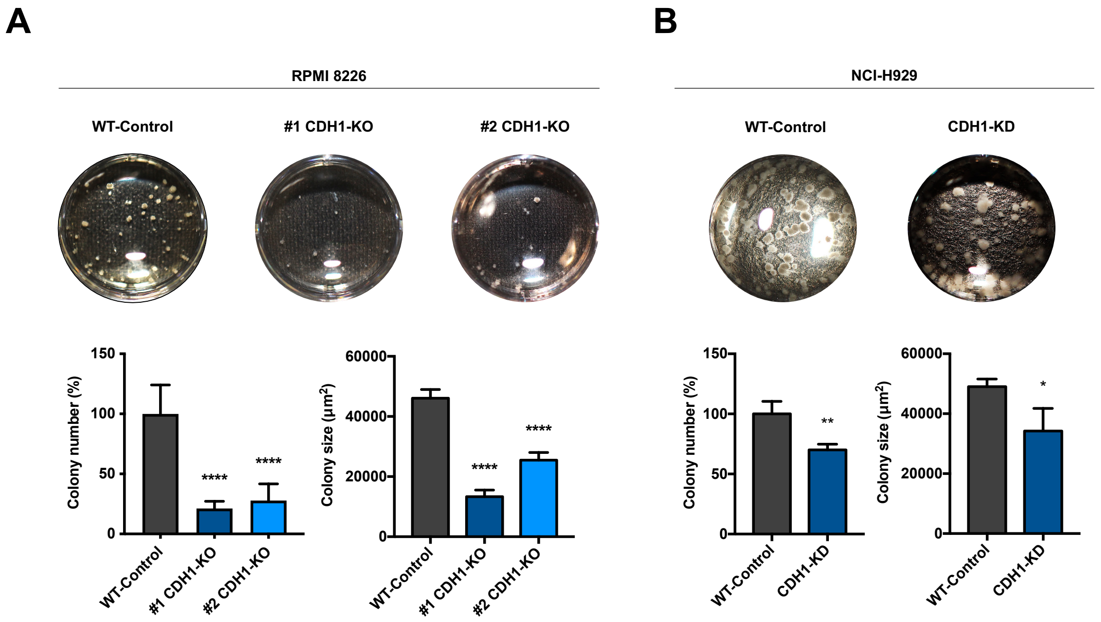

Fig. S3 Depletion E-cadherin inhibits the clonogenic potential of MM cells. **A, B** Clonogenic assay was performed with CDH1-KO RPMI 8226 (**A**) and CDH1-KD NCI-H929 (**B**) cells when compared to WT-control cells. (upper) Representative images of the whole plate are shown. (lower) Percentages of colony number normalized to WT cells (left) and quantitative analysis of colony size (right) in correspond to the data in Fig. 1F. Data are mean ± SD (n = 3). $^{*}p < 0.05$, $^{**}p < 0.01$, $^{****}p < 0.0001$ versus WT cells; two-tailed Student's t-test.

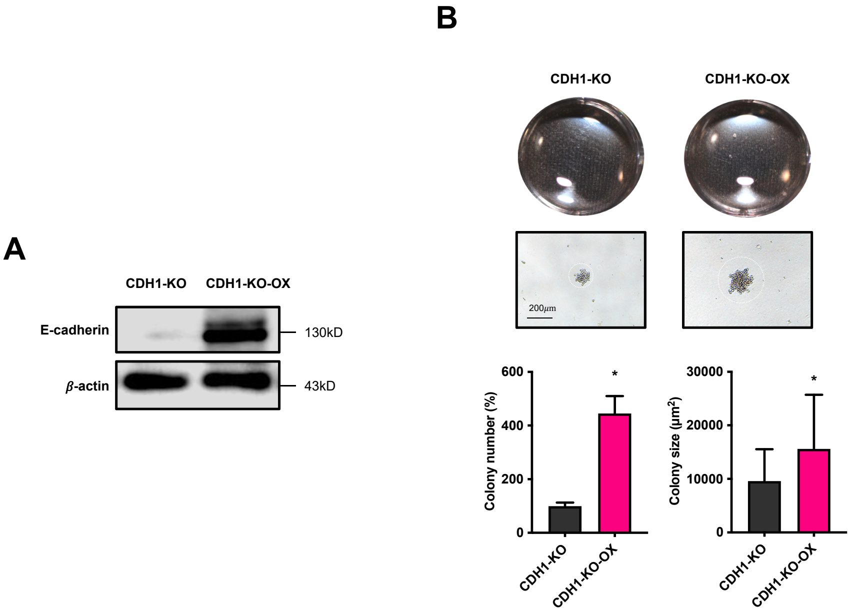

Fig. S4 Restoration of E-cadherin into E-cadherin-depleted cells rescues the clonogenic potential of MM cells. CDH1-KO RPMI 8226 cells were transfected with CDH1 or empty plasmid. Cells with E-cadherin restoration were designated as CDH1-KO-OX. **A** Western blot analysis of E-cadherin level. β-actin was used as a loading control. **B** Analysis of MM colonies under clonogenic assays. (upper) Representative images of plates and micrographs of MM colonies. Scale bar = 200 μm. (lower) Percentages of colony number normalized to CDH1-KO cells (left) and quantitative analysis of colony size (right). $^{*}p < 0.05$ versus CDH1-KO cells; two-tailed Student's *t*-test.

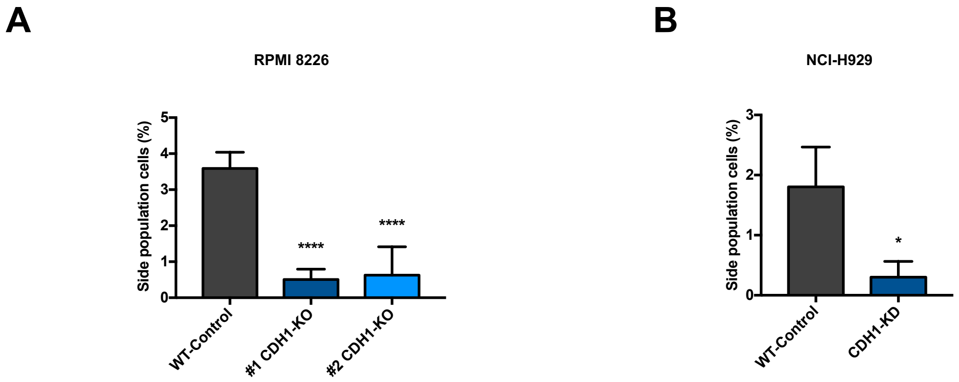

Fig. S5 Depletion of E-cadherin decreases the proportion of SP subpopulation in MM cells. **A, B** Percentages of SP cells in CDH1-KO RPMI 8226 (A) and CDH1-KD NCI-H929 (B) cells comparing to WT control cells in correspond to the data in Fig. 1G. Data are mean ± SD (n = 3). $^{*}p < 0.05$, $^{****}p < 0.0001$ versus WT cells; two-tailed Student's t-test.

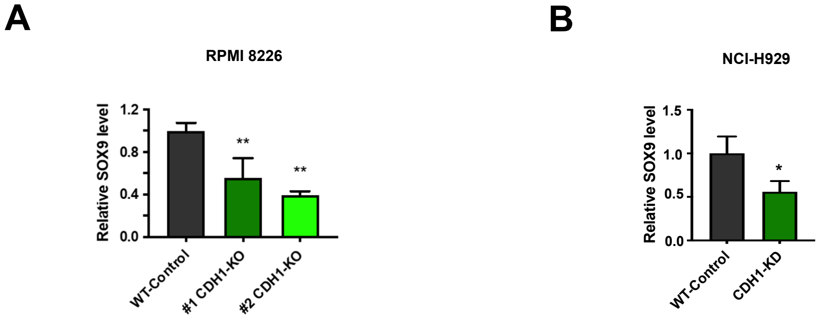

Fig. S6 Depletion of E-cadherin suppresses SOX9 level in MM cells. **A, B** Quantitative analysis of E-cadherin level in CDH1-KO RPMI 8226 (A) and CDH1-KD NCI-H929 (B) cells by densitometry after normalization to β-actin and relative to WT control cells in correspond to immunoblots in Fig. 1J. Data are mean ± SD (n = 3). $^{*}p < 0.05$, $^{**}p < 0.01$ versus WT cells; two-tailed Student's t-test.

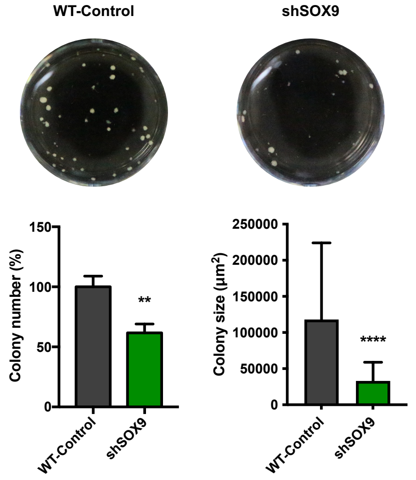

Fig. S7 Depletion of SOX9 reduces the clonogenic potential of MM cells. Clonogenic assay was performed in shSOX9 or empty vector (WT-control) RPMI 8226 cells. (upper) Representative images of the whole plate are shown. (lower) Percentages of colony number normalized to WT cells (left) and quantitative analysis of colony size (right) in correspond to the data in Fig. 2C. Data are mean ± SD (n = 3). $^{**}p < 0.01$, $^{****}p < 0.0001$ versus WT-control cells; two-tailed Student's t-test.

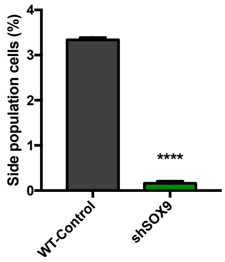

Fig. S8 Depletion of SOX9 decreases the proportion of SP subpopulation in MM cells. Percentages of SP cells in shSOX9 RPMI 8226 cells in correspond to the data in Fig. 2D. Data are mean ± SD (n = 3). $^{****}p < 0.0001$ versus WT-control cells; two-tailed Student's t-test.

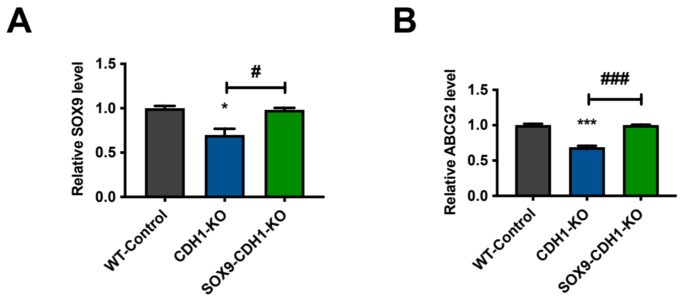

Fig. S9 Re-expression of SOX9 in E-cadherin-depleted MM cells rescues the ABCG2 level. Rescue experiments were performed in CDH1-KO RPMI 8226 cells by transfection of the cells with GFP (CDH1-KO) or SOX9 plasmid (SOX9-CDH1-KO). **A, B** Quantitative analysis of SOX9 (**A**) and ABCG2 (**B**) levels by densitometry after normalization to β-actin and relative to GFP-transfected (WT) control cells in correspond to immunoblots in Fig. 2E. Data are mean ± SD ($n = 3$). $^{*}p < 0.05$, $^{***}p < 0.001$ versus WT cells; $^{\#}p < 0.05$, $^{\#\#\#}p < 0.001$ versus CDH1-KO cells; one-way ANOVA with Tukey's multiple comparison test.

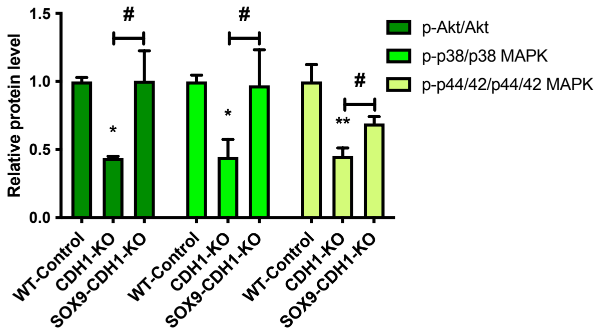

Fig. S10 Re-expression of SOX9 in E-cadherin-depleted MM cells reactivates the Akt and MAPK signaling. Quantitative analysis of p-Akt-to-Akt, p-p38-to-p38 MAPK, p-p44/42-to-p44/42 MAPK ratios in SOX9-CDH1-KO RPMI 8226 cells by densitometry after normalization to β-actin and relative to WT-control RPMI 8226 cells in correspond to immunoblots in Fig. 2F. Data are mean ± SD ($n = 3$). $^{*}p < 0.05$, $^{**}p < 0.01$, versus WT cells; $^{\#}p < 0.05$ versus CDH1-KO cells; one-way ANOVA with Tukey's multiple comparison test.

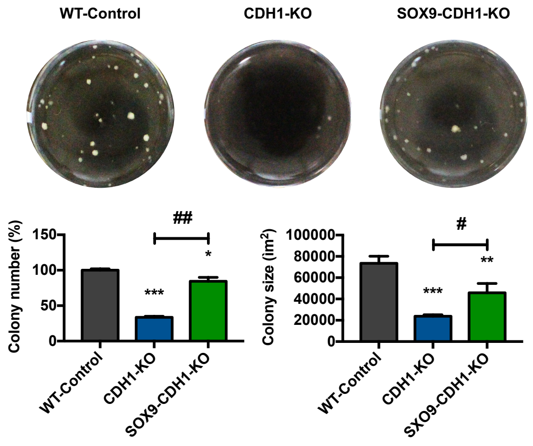

Fig. S11 SOX9 regulates E-cadherin-mediated clonogenic growth in MM cells. Clonogenic assay was performed in SOX9-CDH1-KO RPMI 8226 cells in comparison to WT-control and CDH1-KO cells. (upper) Percentages of colony number normalized to WT cells (left) and quantitative analysis of colony size (right) in correspond to the data in Fig. 2G. Data are mean ± SD (n = 3). $^{*}p < 0.05$, $^{**}p < 0.01$ $^{***}p < 0.001$ versus WT cells; $^{\#}p < 0.05$, $^{\#\#\#}p < 0.001$ versus CDH1-KO cells; one-way ANOVA with Tukey's multiple comparison test.

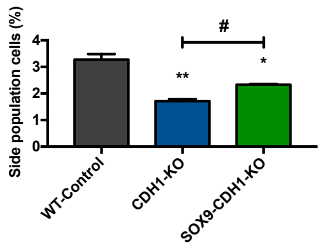

Fig. S12 Re-expression of SOX9 induces the acquisition of SP subpopulation in E-cadherin-depleted MM cells. Percentages of SP cells in SOX9-CDH1-KO RPMI 8226 cells in correspond to the flow cytometric histograms in Fig. 2H. Data are mean ± SD (n = 3). $^{*}p < 0.05$, $^{**}p < 0.01$ versus GFP-transfected-WT-control cells; $^{\#}p < 0.05$ versus GFP-transfected-CDH1-KO cells; one-way ANOVA with Tukey's multiple comparison test.

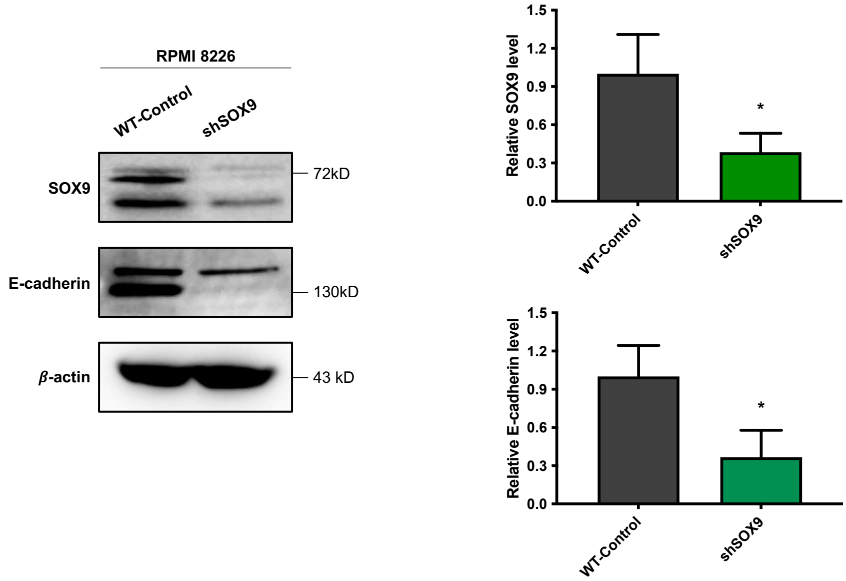

Fig. S13 Inhibition of SOX9 suppresses E-cadherin level in human MM-derived cells. SOX9 was depleted in RPMI 8226 cells using lentiviral particles carrying shSOX9 or non-target sequence (WT-control), and E-cadherin level was evaluated by Western blotting. (left) Representative immunoblots are shown. (right) Quantitative analysis using densitometry, after normalization to β-actin. Data are mean ± SD ($n = 3$). $^{*}p < 0.05$ versus WT cells.
